# Unpacking the Development of Chinese Preservice English as a Foreign Language Teachers’ Professional Knowledge

**DOI:** 10.3389/fpsyg.2022.883056

**Published:** 2022-06-09

**Authors:** Liyan Liu, Anne Li Jiang, Shiyu Yang, Shuo Li

**Affiliations:** ^1^School of Foreign Languages, Northeast Normal University, Changchun, China; ^2^Faculty of Education, Northeast Normal University, Changchun, China

**Keywords:** preservice EFL teachers, professional knowledge development, PK, CK, PCK

## Abstract

Efforts to improve preservice teacher education have recently focused on developing teachers’ adequate pedagogical knowledge (PK), content knowledge (CK), and pedagogical content knowledge (PCK), which are critical elements of teacher’s professional knowledge, and important indicators of preparedness to teach. However, the development of the three knowledge domains of Chinese preservice English as a foreign language (EFL) teachers is surprisingly under-researched. To fill this gap, this study examined the development of the three knowledge domains of a group of Chinese preservice EFL teachers at different stages of a teacher education program. Specifically, it explored the relationship among the three knowledge domains, and the effects of learning opportunities on their development. Findings revealed that preservice EFL teachers at a later stage outperformed those at an earlier stage with regard to PK and PCK. Our findings also suggested that there were positive correlations among PK, CK, and PCK at different stages of the teacher education program. Furthermore, the findings showed that courses on CK, PK, and PCK, and teaching experience significantly influenced preservice EFL teachers’ professional knowledge. However, the role of classroom observation was not significant. Implications for EFL teacher education and future research were also discussed.

## Introduction

Teaching is not simply transmitting information but a complex process that requires teachers to apply multiple domains of knowledge to facilitate students’ understanding (Wilson et al., 1987; Park and Chen, 2012). To professionalize the complex act of teaching, teachers need to develop a special body of knowledge that exceeds content knowledge. Therefore, teachers’ professional knowledge was proposed in the field of teacher education (Shulman, 1987). Though teachers are expected to have an adequate command of all professional knowledge domains, three knowledge domains, namely, pedagogical content knowledge (PCK), content knowledge (CK), and pedagogical knowledge (PK) are believed to be critical for teachers to create high-quality instruction (Baumert et al., 2010; König et al., 2016; Evens et al., 2018; Sorge et al., 2019). It is therefore an important task for teacher education programs to facilitate preservice teachers’ development of CK, PK, and PCK.

Research has continuously attested to the necessity for teacher education programs to cultivate preservice teachers’ CK, PK, and PCK in order to facilitate their sustainable development (Kulgemeyer and Riese, 2018; Brandt et al., 2019). Traditional teacher education programs usually treat these knowledge domains separately. However, emerging arguments and evidence have shown that the development of CK, PK, and PCK was deeply intertwined (Gess-Newsome et al., 2017; Sorge et al., 2019). The possession of adequate CK is a prerequisite for teachers to develop effective PCK (Smith and Banilower, 2015). PK also plays a vital role in creating and optimizing teaching situations to transmit subject matter (Voss et al., 2011). Therefore, qualified teachers are expected to be able to integrate CK, PK, and PCK. Recently, there has been a growing interest in conducting research on examining the interplay among these three knowledge areas (Großschedl et al., 2015; König et al., 2016; Evens et al., 2017). Of the few attempts to delineate the interplay, most were carried out in the science and mathematics domains. However, how CK, PK, and PCK interact with one another in the English as a foreign language (EFL) teaching context has not been fully resolved. Teaching EFL is distinct from the teaching of other subjects for various reasons, among which the most important one is that the content of language teaching and the medium of instruction are the same (Evens et al., 2016; König et al., 2016). Given the unique feature of EFL teaching, any attempt to explore how EFL preservice teachers’ PK, CK, and PCK interact with each other would undoubtedly be necessary.

Moreover, research has shown that preservice teachers usually feel challenged to integrate the different knowledge types individually (Harr et al., 2015) and the literature highlights that learning opportunities (e.g., teacher education courses, teaching practice, and classroom observation) provided in teacher education programs can enhance preservice teachers’ sustainable knowledge development (Evens et al., 2017, 2018; Bürgener and Barth, 2018; Brandt et al., 2019). However, there is a lack of research on how various learning opportunities affect the development of their professional knowledge. Especially, the specific contribution of each of these learning opportunities to the development of PK, CK, and PCK remains unclear until recently. Therefore, further research is needed regarding the development of the interplay among EFL preservice teachers’ PK, CK, and PCK, and regarding how various learning opportunities provided in the program affect that development. As such, this study seeks to answer the following research questions:

1. What are the levels of PK, CK, and PCK of the Chinese preservice EFL teachers at different stages of the teacher education program?
2. How does the relationship among PK, CK, and PCK of the Chinese preservice EFL teachers change along with the progress of the program?
3. How do learning opportunities, namely, courses on PK, CK, and PCK, classroom observation, and teaching experience, influence the Chinese preservice EFL teachers’ professional knowledge?

## Literature Review

### Conceptual Framework of Teachers’ Professional Knowledge

The research on professional knowledge is mostly inspired by the study of Shulman (1986, 1987). In 1987, he proposed seven categories of teacher knowledge base which consisted of content knowledge (CK), general pedagogical knowledge (GPK/PK), pedagogical content knowledge (PCK), curriculum knowledge, knowledge of learners, knowledge of educational context, and knowledge of educational ends, purposes, and values (Shulman, 1987). Among these knowledge bases, the first three components were widely considered as the core of professional knowledge in subsequent research (Grossman, 1990; Paulick et al., 2016; Neubrand, 2018).

One of the significant contributions of Shulman (1987) in teacher knowledge studies is to emphasize the role of content in teaching. The first domain of content-related knowledge is CK, which originally referred to knowledge and disposition that should be learned by students, the knowledge of the subject and its organizing structure (Ball et al., 2008). Teaching a subject requires more than knowing the concepts and facts in a specific domain. It is necessary for teachers to further understand the reasons, principles, scopes of application, and position (whether something is central or peripheral in a discipline) of the knowledge (Shulman, 1986). There is a growing consensus in teacher knowledge literature that CK contributes to teaching quality and students achievement (Baumert et al., 2010) and it is a prerequisite for PCK development (Magnusson et al., 1999; Kleickmann et al., 2013). According to Baumert et al. (2010), CK is proved to have predictive power for student mathematics achievement as well as high correlation with PCK, defining the possible scope for the improvement of PCK. Diverse instruments to assess CK have been developed in different disciplines, namely in mathematics (Hill, 2007), physics (Sorge et al., 2019), biology (Großschedl et al., 2015), and English (König et al., 2016). For instance, CK in physics comprises the following content areas: mechanics, optics, electricity, and solid state physics, etc. (Paulick et al., 2016). Instruments of CK in English mainly measures knowledge of American and British literature and linguistics (König et al., 2016).

Although many researchers agree that CK should be positioned at a crucial role in teaching (Ball et al., 2008; Baumert et al., 2010; Kleickmann et al., 2013), it also has been recognized that CK alone is far from sufficient for effective teaching and learning (Baumert et al., 2010; Yang et al., 2020). Knowing a lot of the content does not mean knowing how to make the content accessible to students. PCK, the second domain of content-related knowledge, is more positively related to teachers’ instructional practice. Defined by Shulman (1986, 1987) as the special amalgam of content and pedagogy, PCK represents a hybrid of discipline-based content knowledge and training-based pedagogical knowledge into an understanding of how to organize and represent a particular topic and makes it comprehensible to students. Gess-Newsome (2015, p. 31) extended its connotation by redefining it in The PCK Summit as “both a knowledge base used in planning for and the delivery of topic-specific instruction in a very specific classroom context, and as a skill when involved in the act of teaching.” It can be seen that PCK is nowadays considered as highly topic- and context-specific. Inspired by Shulman’s work, different researchers demonstrated their understandings on PCK in terms of its components. Initially, two facets of PCK were identified, namely knowledge of instructional strategies and knowledge of students’ understanding. The former one referred to the knowledge of how teachers represented subject matter and made it comprehensible for students. The latter one included the knowledge of students’ preconceptions (Shulman, 1986). Grossman (1990) extended this concept by conceptualizing PCK as a model constituting four components: (i) conceptions of purposes for teaching subject matter, (ii) knowledge of students’ understandings, (iii) curricular knowledge, and (iv) knowledge of instructional strategies. Building on this, Magnusson et al. (1999) added one component in PCK model for science teaching, which is knowledge of assessment of scientific literacy. In this model, orientation to teaching subject matter shapes and interacts with other components of PCK. The present study followed the initial research and considered PCK as a two-facet model.

In contrast to CK and PCK, PK refers to “broad principles and strategies of classroom management and organization” (Shulman, 1987, p. 8), which transcends subject matter. Grossman and Richert (1988) extended this definition by including four domains in their conceptualization of PK: (i) knowledge of theories of learning and general principles of instruction, (ii) understanding of the various philosophies of education, (iii) general knowledge about learners, and (iv) knowledge of principles and techniques of classroom management. König et al. (2011) identified four dimensions of PK, including “structure” (i.e., plan, structure, and evaluate lessons), “motivation and classroom management” (i.e., engage students and organize classroom), “adaptivity” (i.e., adapt to student heterogeneity), and “assessment” (i.e., evaluate students with diverse assessment types and criteria). In a sample of 746 German teacher candidates, Voss et al. (2011) noticed a positive correlation between levels of PK and instructional quality rated by students.

Emerging arguments and evidence suggest the deeply intertwined and codependent relationship between CK and PCK, which was also illustrated by the integrative model and transformative model proposed by Gess-Newsome (1999). In the integrative model, PCK is not a separate category of knowledge but a dynamic interaction and overlap of subject matter knowledge (SMK), pedagogical knowledge, and contextual knowledge. In the transformative model, PCK represents a transformation of subject matter knowledge and other knowledge bases into unique knowledge for the purposes of effective instruction. The fundamental difference between these two models lies in whether CK is a separate knowledge category or not (Gess-Newsome, 1999). Empirical research conducted by Kramer et al. (2021) also confirmed that CK and PCK were moderately correlated. While expansive body of research on teacher professional knowledge has largely been based on CK and PCK over the past 30 years, PK was sometimes neglected and it has only been investigated in recent years (König et al., 2011, 2016; Großschedl et al., 2015; Evens et al., 2017; Sorge et al., 2019). In addition, little attention was devoted to examining the interrelation and development of CK, PK, and PCK over time. The interplay of the three components of professional knowledge in EFL teaching has not been thoroughly investigated (König et al., 2016), which will be one of the focuses of the present research.

### Development of Teachers’ Professional Knowledge and Its Influencing Factors

Grossman (1990) identified the following four different sources for the construction and development of teacher knowledge: (i) apprenticeship of observation (which mostly benefits PCK and curriculum knowledge), (ii) subject matter knowledge (which helps teachers choose, judge, and arrange the subject content critically), (iii) teacher education (which mainly provides educational courses and contributes to the construction and development of PCK by covering the subject structure, fundamental teaching ideas, and specific teaching techniques, etc.), and (iv) actual teaching experience (which offers classroom to test and improve their acquired knowledge). Those resources were supported and developed by later research. Adopting qualitative research method, Lawrie et al. (2018) investigated chemistry teachers’ perception of their professional knowledge development with the influencing factors. The results indicated that the development of professional knowledge was built on mentorship, depth of curriculum and content knowledge, depth of teachers’ experience, and purposeful reflection. Van Driel and Berry (2012) stated that collaboration, collegiality, and the fostering of relationships were also helpful.

For preservice teachers, teacher education institutions offer a variety of learning opportunities for them to acquire and develop professional knowledge. Cross-sectional studies in European countries pointed out that differences between the development of preservice teachers’ professional knowledge were accounted for by differential learning opportunities throughout teacher education (Großschedl et al., 2015; König et al., 2016; Evens et al., 2017; Sorge et al., 2019). For instance, Großschedl et al. (2015) measured the relationship of CK, PK, and PCK of 274 German biology preservice teachers and the effects of learning opportunities on professional knowledge in terms of four aspects: (i) types of teacher educational program (academic track or non-academic track), (ii) period of university studies (locate at university or teaching training school), (iii) second teaching subject (science subject or human/social science subject), and (iv) teaching experience (the number of lessons they taught). The results showed that there was a positive correlation between PCK and CK, as well as PCK and PK. Academic-track participants outperformed nonacademic-track ones on CK and PCK. Preservice biology teachers who were in the later period of studies performed better on CK and PCK. In addition, a second science subject and longer teaching experience positively correlated with PCK performance. In the domain of physics in Germany, Sorge et al. (2019) noticed a remarkable shift of the interplay of components in professional knowledge across different stages, and the significant impacts of the number of terms and the amount of classroom observation on the components in teacher education programs.

In the context of Belgium with different teacher education programs and tradition in contrast to Germany, Evens et al. (2017) compared the professional knowledge of three cohorts of preservice teachers (from the first year to the third year) who were prepared to become generalists in primary school. Four types of learning opportunities were considered to account for the impact on professional knowledge: the number of course hours on CK, PK, and PCK in French, and the number of internship days. They found that preservice teachers performed better in the second and third year in comparison with those in the first year. Courses on PCK have positive effects on preservice teachers’ PCK and PK while practical experience did not show significant effects on any knowledge domain.

The first attempt to investigate the structure and development of preservice teachers’ professional knowledge in the domain of English was made by König et al. (2016) to our knowledge. They stated the unique characteristics of teaching English as a foreign language (TEFL) and drew the conclusion that professional knowledge on TEFL was a multidimensional construct and PCK correlated closely both with CK and PK. With regard to the influence of learning opportunities, namely teacher education program and phases in this research, future lower/upper secondary teachers outperformed Lower secondary teachers in terms of CK and PCK. Preservice teachers at a late stage (practical phase) performed better than those at an early stage at university (theoretical phase) in PK and PCK.

To sum up, most of the previous research is situated in the domain of science and mathematics while empirical research in EFL teaching is rare (König et al., 2016). In addition, how preservice teachers’ professional knowledge was shaped by teacher programs in European counties has been thoroughly discussed. However, whether these findings can be applied in different contexts and teacher education programs waits to be proved. Since the development of professional knowledge is a complex process and specific to situation, context, and individuals, it is necessary to reconsider the professional knowledge for EFL teaching in Chinese educational context. The knowledge gap that the present research tries to fill concerns examining the change of the interplay of preservice EFL teachers’ PK, CK, and PCK in professional knowledge in Chinese context as well as the impacts of learning opportunities in teacher education programs on the development of preservice EFL teachers’ professional knowledge.

## Materials and Methods

### Context

The past decade in China has witnessed a national drive to reform and modernize its educational system. In 2011, the Chinese government published its *Teacher Education Curriculum Standards* (Ministry of Education China, 2011). Like all other countries that are seeking ways to improve the quality of their teachers, China is trying to develop pedagogy for teacher education that can effectively link theory to practice (Korthagen et al., 2006). The expectation for teachers to become change agents (Lo, 2009) as part of the reform efforts is clear.

In such a macro context, the present study was set in X university, a famous normal university in Northeast China. To cultivate excellent EFL teachers for secondary schools, the 4-year undergraduate teacher education program in X University adopts a “2 + 1 + 0.5 + 0.5 U-G-S” collaborative practicum model. Here “2” refers to the first 2 years of basic course learning when core knowledge domains of CK and PK are delivered across courses. “1”represents the third year of learning that focuses more on specialized CK and PCK, in which preservice teachers have 2 days to observe classroom teaching every month during their observational visits in local secondary schools. The first “0.5” stands for the first semester of the fourth year during which preservice teachers have 2 months’ residency practicum at designated partner secondary schools under the collaborative supervision of university teachers and school mentors supported by the local government. This is called the “university-government-school” (U-G-S) model, which provides preservice teachers with plenty of opportunities to observe mentor teachers’ classroom teaching and to have hands-on experiences of working as real school teachers under the collaborative supervision of both school mentors and university teachers. The second “0.5″ represents the second semester of the fourth year, which mainly emphasizes preservice teachers’ reflection on educational practice and thesis writing. Following this full cycle of learning, experiencing, teaching, and reflecting, preservice teachers are encouraged to integrate CK, PK, and PCK in order to enhance their ability to create quality instruction. The courses involved in the program were taught by the same body of university teachers, meaning a specific course is taught by the same teacher or the same group of teachers collaboratively. Therefore, the participants of this study were taught by the same teacher cohort. Overall, the program is designed as such to develop preservice teachers’ professional knowledge through close integration of undergraduate coursework and teaching practicum in partner schools.

### Participants

In total, 315 preservice EFL teachers from Year 2 to 4 of the teacher education program in X University were involved as participants. Among them, 100 were Year 2 participants with an average age of 19.5 years (*SD* = 3.52; 10% male), 119 were Year 3 participants with an average age of 20.56 years (*SD* = 3.21; 12.6% male), and 96 were Year 4 participants with an average age of 22.1 years (*SD* = 2.89; 9.3% male). Participation was based on informed written consent. Ethics approval was obtained from the authors’ university. Since these participants had no teaching experience in middle schools, they were only tested on CK upon entering the program. Then the scores of the three cohorts of participants, i.e., Year 2, Year 3, and Year 4 students, were compared. As shown in Table 1, the results of one-way ANOVA analysis showed that there was no significant difference in the baseline level of the three cohorts’ CK (*F* = 10.22, *p* > 0.05).

**Table 1:** Comparison among three preservice teacher cohorts’ content knowledge (CK) scores.

Cohorts	CK			*F*
	*N*	*M*	*SD*	
Year 2	88	35.62	6.38	10.22
Year 3	115	34.83	5.92	
Year 4	90	36.21	8.99	

### Instrument

To assess preservice EFL teachers’ professional knowledge, a standardized paper-and-pencil test was developed based on work of König et al. (2011), which is intended to test preservice EFL teachers’ CK, PCK, and PK. All the items of our test were developed by experts and researchers of education, psychology and English education drawing on the *Teacher Education Curriculum Standards* (Ministry of Education China, 2011), Chinese preservice EFL teachers’ qualification examination, and *General Senior High School English Curriculum Standards* (Ministry of Education China, 2017).

The CK test mainly examines preservice EFL teachers’ comprehensive English language knowledge, English and American literature, and cross-culture knowledge. This part contains 40 multiple-choice questions. The test of PCK focuses on two aspects: knowledge of instructional strategies and knowledge of students’ understanding. There are four open-ended questions. The test of PK aims to assess four aspects of knowledge which contains 15 multiple-choice questions and five short answer questions. It includes broad principles and strategies of teaching structure, classroom management, adaptivity, and assessment (See Appendix for the example questions).

To ensure the reliability of the test, rigorous process of expert ratings, guided interviews and extensive piloting of the items were followed. Firstly, inter-rater reliability was calculated. Cohen’s *k* of 0.85 for PK, 0.84 for PCK, and 0.76 for CK were found, indicating good agreement for CK and very good agreement for PK and PCK. Secondly, to measure internal consistency, Cronbach’s *α* was calculated. The reliability statistics show good internal consistency of the PCK, CK, and PCK tests (PCK *α* = 0.73; PK *α* = 0.74; and CK *α* = 0.88).

### Data Collection

The dataset of this study consisted of a paper-and-pencil test and documents as well as materials including the education program policy, course syllabus, and preservice teachers’ reflective journals.

First, the paper-and-pencil test used to measure preservice EFL teachers’ professional knowledge (i.e., CK, PCK, and PK) were administrated, respectively, to the sophomores, juniors, and seniors at the end of Year 2, Year 3, and Year 4. Prior to the test administration, the research purpose, the structure of professional knowledge, and the test format were introduced and explained to the participants, and the participants had 2 h to complete the test with two 10-min breaks. Around 315 test papers were collected in total, with a response rate of 100%.

Apart from the test, we also collected related program documents such as the program policy, course syllabus, teaching schedule, etc. In order to better understand the perceived influence of learning opportunities on preservice EFL teachers’ professional knowledge development; we also collected the reflective journals of 12 voluntary preservice teachers from Year 3 to Year 4 participants at the end of their respective school year.

### Data Analysis

To analyze the test data, we first put participants’ answers into a data file. Multiple-choice items were scored full credit (1 point) or no credit (0 points), short answer and open-ended items were scored as partial credit (typically 0, 1, or 2 points depending on the quantity and quality of the answers). To assure the reliability of the scoring for short answer and open-ended items, a sample of approximately 20% of the items were scored by a second rater. The interrater reliability was calculated using Cohen’s kappa, which showed good agreement between both raters, specifically 77% for the PCK items, 78% for the CK items, and 73% for the PK items. Statistical analysis was then performed. Descriptive statistics and Pearson correlation were used to analyze the levels and correlations of participants’ CK, PK, and PCK at different learning stages of the program. Multiple regression analysis was performed to reveal the impact of learning opportunities on the teachers’ professional knowledge.

In addition, the curriculum syllabus, course timetables, the preservice EFL teachers’ reflective journals of their school visits, and practicum were also analyzed to obtain information about their learning opportunities and their perceptions on the influence of the learning opportunities. Qualitative thematic analysis was employed to analyze the journal entries (Miles et al., 2014). These journals were read and reviewed several times carefully to identify the themes concerning how learning opportunities influenced preservice teachers’ professional knowledge development throughout the program. These themes were constantly compared and modified within and across these 12 participants to reveal similarities and differences. To ensure trustworthiness, coding was also conducted by two researchers with a high inter-rater reliability (Cohen’s *K* > 0.8).

## Findings

### The Levels of PK, CK, and PCK

With our first research question, we intended to explain the levels of professional knowledge of Chinese preservice EFL teachers in terms of different stages of teacher education programs. Table 2 shows the descriptive statistics related to preservice EFL teachers’ professional knowledge. With regard to the general professional knowledge, Year 3 participants had the highest test scores. Specifically, preservice EFL teachers at a later stage outperformed those at an earlier stage in terms of PK and PCK, which indicated a sustainable development of PK and PCK during the whole teacher education programs. Concerning CK, the mean score in the third year is the highest among these three groups.

**Table 2:** Descriptive statistics related to CK, pedagogical knowledge (PK), and pedagogical content knowledge (PCK) scores.

Cohort	CK				PK			PCK			Professional knowledge		
	*N*	*M*	*SE*	*SD*	*M*	*SE*	*SD*	*M*	*SE*	*SD*	*M*	*SE*	*SD*
Year 2	88	40.33	0.78	7.27	10.97	0.26	2.47	3.07	0.18	1.70	54.27	0.92	8.69
Year 3	115	42.22	0.60	6.40	12.10	0.23	2.52	5.03	0.26	2.79	59.27	0.78	8.39
Year 4	90	39.27	0.95	9.01	12.38	0.28	2.71	7.34	0.32	3.01	58.92	1.24	11.81
Overall	293	40.74	0.44	7.62	11.84	0.15	2.62	5.15	0.18	3.07	57.66	0.57	7.49

### The Relationship Among PK, CK, and PCK

Regarding our second research question, we intended to explore the relationship among PK, CK, and PCK of preservice EFL teachers’ professional knowledge. The correlations among these three domains were presented in Table 3. We found a positive correlation between CK and PCK in Year 2 and Year 4 participants, which shifted from 0.32, *p* < 0.01 to 0.27, *p* < 0.01. There was a positive correlation between CK and PK, increasing from 0.27, *p* < 0.01 in Year 3 to 0.56, *p* < 0.01 in Year 4, which indicated that more advanced preservice EFL teachers integrate CK and PK better. We also observed a closely correlation between PK and PCK in Year 4 participants of the teacher education program.

**Table 3:** Correlation among three dimensions of professional knowledge for the second year to fourth year preservice English as a foreign language (EFL) teachers.

	Year 2			Year 3			Year 4		
	CK	PK	PCK	CK	PK	PCK	CK	PK	PCK
CK	-			-			-		
PK	0.11	-		0.27^**^	-		0.56^**^	-	
PCK	0.32^**^	0.06	-	0.12	0.18	-	0.27^*^	0.33^*^	-

** *p* < 0.01;

* *p* < 0.05.

### The Influences of Learning Opportunities on PK, CK, and PCK

The third research question aims to investigate the effects of learning opportunities on the three domains of professional knowledge. In Table 4, an overview of learning opportunities in the participating university was displayed. In all the three cohorts, X university provided a larger number of CK courses in comparison with PK and PCK courses. No teaching experience and PCK courses were provided in the second year.

**Table 4:** Number of learning opportunities concerning PCK, PK, and CK.

	Courses on PK (hours)	Courses on CK (hours)	Courses on PCK (hours)	Classroom observation (hours)	Teaching experience (days)
Year 2	96	432	0	6	0
Year 3	120	720	168	60	12
Year 4	144	792	216	120	72

Concerning PK, the model including courses on PK and teaching experience as predictors explained 17.3% of the variance of PK, *F* = 5.19, *p* < 0.05. Table 5 presented the regression coefficients. Courses on PK positively influenced preservice EFL teachers’ PK (*β* = 0.002, *p* = 0.002). Furthermore, the preservice EFL teacher with more teaching experience performed better on PK (*β* = 0.005, *p* = 0.003).

**Table 5:** Results of multiple regression analyses with PK as criterion.

Model	*B*	*SE*	*t*	*p*
Constant	4.835	0.508	9.513	0
Gender	−0.117	0.193	−0.605	0.545
Course on PK	0.002	0.001	3.125	0.002
Teaching experience	0.005	0.003	0.008	0.003

Regarding CK, the model containing courses on CK as predictor explained 23% of the variance of CK, *F* = 2.73, *p* < 0.05. Table 6 showed the regression coefficients. The courses on CK significantly impacted CK (*β* = 0.057, *p* = 0.005).

**Table 6:** Results of multiple regression analyses with CK as criterion.

Model	*B*	*SE*	*t*	*p*
Constant	36.85	2.984	12.349	0
Gender	0.38	1.133	0.335	0.738
Course on CK	0.057	0.02	2.847	0.005

With regard to PCK, the model included courses on CK, courses on PCK, and teaching experience as predictors, explaining 29.7% of the PCK variance (*F* = 40.768, *p* < 0.001). The regression coefficients were presented in Table 7. Both courses on CK (*β* = 0.007, *p* = 0.000) and courses on PCK (*β* = 0.22, *p* = 0.000) showed significant effects on PCK. In addition, teaching experience positively predicted the development of PCK (*β* = 0.03, *p* = 0.000). Concluding the analysis, it was important to note that courses on CK, PK, and PCK, and teaching experience were the main sources of preservice EFL teachers’ professional knowledge (Table 7).

**Table 7:** Results of multiple regression analyses with PCK as criterion.

Model	B	SE	t	p
Constant	−0.454	1.024	−0.444	0.658
Gender	0.336	0.389	0.863	0.389
Courses on CK	0.007	0.001	5.311	0.000
Courses on PCK	0.22	0.342	4.336	0.000
Teaching experience	0.03	0.007	4.388	0.000

The analysis of Year 3 participants’ reflective journals concerning their lesson observation during the school visits revealed the following issues that may affect their knowledge improvement. First, as there was a lack of effective supervision from teacher educators, preservice EFL teachers were confused about what to observe in classroom and how to give valid reflection since teacher educators did not cover that in teacher education courses, as indicated in the following quotes,

Excerpt 1 (S8-reflective journal on school visits)

*I did not enjoy my school visits, because I really did not know what to focus on when sitting in the classroom, and nobody told me what to do before and after the classroom observation.*

Second, misalignment seemed to exist between the observed and the learnt teaching philosophies. One participant wrote in the journal,

Excerpt 2 (S11-reflective journal on school visits)

*The design of classroom activities did not reflect the English teaching activity theory mentioned in the curriculum standards. It was contrary to what I learned from the university courses, such as curriculum standard analysis.*

From the journals of Year 4 participants, two aspects particularly conducive to knowledge improvement were identified. For one thing, students reflected the co-supervision by university teachers and middle school mentors were quite helpful for the development of their PCK.

Excerpt 3 (S3-reflective journal on school practicum)

*Under the collaborative guidance, the teaching experience gave me good understanding of students’ learning difficulties, interest and developmental level, and that understanding probably facilitate me to better recognize students’ needs and sequence proper activities to motivate students’ participation in learning.*

For another, collaborative peer lesson planning and rehearsal teaching effectively promoted participants’ reflection on educational theoretical knowledge, as one student wrote,

Excerpt 4 (S6-reflective journal on school practicum)

*The peer discussion before the real teaching helped me to rethink the general principles and guidelines I had learned, and I got better understanding of how to reuse the principles and guidelines according to the real classroom teaching context.*

## Discussion

The three knowledge dimensions of PK, CK, and PCK are believed to be critical for teachers to create high-quality instruction (Baumert et al., 2010; König et al., 2016; Evens et al., 2017; Sorge et al., 2019). Research has continuously attested to the necessity to explicitly provide all three knowledge domains of CK, PK, and PCK in teacher education programs in order to secure preservice teachers’ sustainable development (Kulgemeyer and Riese, 2018; Brandt et al., 2019). Through examining the development of PK, CK, and PCK of a group of Chinese preservice EFL teachers at different stages of a teacher education program, the present study found that there was improvement in preservice EFL teachers’ PK and PCK along with the progress of the program. Year 3 participants had the highest CK scores. In addition, positive correlation was found among CK, PK, and PCK at different stages. Courses on professional knowledge and teaching experience significantly predicted the development of preservice EFL teachers’ professional knowledge. Surprisingly, classroom observation had no effects on either domain of professional knowledge.

Concerning the levels of PK, CK, and PCK, relatively significant differences were found at different stages of the program. Older preservice teachers performed significantly better on PK and PCK, which corresponds to the result of König et al. (2016). According to König et al. (2016), preservice EFL teachers at a later stage (practical phase) performed better than those at an earlier stage at university (theoretical phase) on PK and PCK. Our findings thus attested to the positive role of teacher education for the sustainable development of PK and PCK. However, no significant development was found in terms of CK from the early to the late stages, and Year 3 participants’ CK performance was the best. This finding could be explained by the fact that, in X University, Year 3 participants had more CK courses than Year 2 and Year 4 participants did. The main CK courses offered in Year 3 are linguistics, cross-cultural communication, and appreciation of literary works, which are crucial for preservice EFL teachers to develop their CK.

Regarding the relationship between the three knowledge domains, we found a positive correlation between CK and PCK in Year 2 and Year 4 participants, indicating that preservice EFL teachers with higher CK also tend to have higher PCK, which is in line with the results of previous studies (Magnusson et al., 1999; Baumert et al., 2010; Kleickmann et al., 2013). In addition, the present study found the correlation between CK and PCK was much higher in Year 2 (*r* = 0.32, *p* < 0.01) than in Year 4 (*r* = 0.27, p < 0.01). A plausible reason for this result is that in X University, Year 2 participants have more learning opportunities on CK than Year 4 participants. It is widely acknowledged that CK is a critical foundation of PCK and can transform into PCK with other knowledge bases (Shulman, 1987; Gess-Newsome, 1999). Since Year 2 participants attend more courses on CK, they are prone to have higher CK, which in turn is liable to be transformed into PCK. Furthermore, a positive correlation between CK and PK in the third year was found, which indicates that a high performance in CK is accompanied with a high performance in PK. As a knowledge base, CK was closely and positively related to PK and PCK in the fourth year, which supports the importance of CK for the development of PK and PCK. Finally, PK and PCK were correlated in the fourth year, which is in line with the results of Großschedl et al. (2015) and König et al. (2016). This correlation attests to the fact that teaching practice can greatly promote the integration and PK and PCK.

As to the influence of learning opportunities on the development of preservice EFL teachers’ professional knowledge, X University offered a variety of learning opportunities for them to acquire and develop professional knowledge. The present study suggested that the courses on PK, CK, and PCK, which were improvised in the first 3 years, and the teaching practicum during the first semester of the fourth year were positively related to participants’ professional knowledge development.

Regarding the effects of PK, CK, and PCK courses, the present study found that PK courses was a positive predictor of the development of PK. CK courses had a significant impact on CK and PCK. PK and PCK courses were positively related to preservice EFL teachers’ PK and PCK, respectively. The positive effect of CK courses on CK development was in line with previous research in mathematics (Blömeke et al., 2012; Qian and Youngs, 2015) and in French as a foreign language (Evens et al., 2017). Additionally, the finding that the more PCK courses preservice EFL teachers had, the better their test performance on PCK confirmed the previous research that has found positive effect of PCK courses on PCK (Blömeke et al., 2012; Qian and Youngs, 2015; Evens et al., 2017; Sorge et al., 2019).

With regard to the effect of teaching practicum, the present study affirmed the importance of teaching experience in teacher education. To be specific, the result has shown that the more teaching experiences that preservice EFL teachers had, the better they performed in the PK and PCK, which indicates that teaching experience is crucial to the development of PK and PCK. However, this is inconsistent with Evens et al. (2017) and Sorge et al. (2019) who found teaching experience did not significantly impact the three knowledge domains. Evens et al. (2017) ascribed their result to the fact that the internship was too short to develop preservice teachers’ professional knowledge. Nevertheless, X University provides preservice EFL teachers with 2-month residency practicum under the collaborative guidance of university supervisors and school mentors, who will work together to facilitate preservice teachers to make improvement. The immediate and constructive feedback and group discussions helped preservice teachers digest and internalize what they learned from university courses and to better learn new methods and representations.

Finally, it was surprising that no significant effect of classroom observation on all the three domains of professional knowledge was found. This finding did not match the result of Sorge et al. (2019) who pointed out that classroom observation and reflections can support the development of all the three domains and hence enable preservice physics teachers to gain an integrated understanding on the planning and enactment of teaching. Two possible reasons may explain the findings of the present study. From what is reflected in participants’ reflective journals on their class observation, it can be seen that the effect of this learning opportunity is undermined by the lack of guidance and supervision from teacher educators, and the mismatch between the learned theory and observed teaching behavior. Another reason is that the observation opportunity is too limited as the Year 3 participants only had it once a month for 4 months, which is insufficient to support the development of their professional knowledge.

## Conclusion and Implication

Our findings suggested that sustainable development of preservice EFL teachers’ professional knowledge is realized during the teacher education program. The three domains, namely CK, PK, and PCK, were deeply intertwined and inter-dependent with each other at different stages of the teacher education program. Both universities courses and teaching experience are conducive to the development of the three knowledge domains of professional knowledge. Given that most extant empirical studies of this line of inquiry focus on other disciplines like mathematics, physics, biology, and French as a foreign language, this study is among the few that sheds light on the developmental trajectories of preservice teachers’ professional knowledge in the EFL discipline, and thus enriches the literature on preservice teacher education and professional development.

This study has obvious limitations. Firstly, the sample is homogeneous in the sense that only one university was involved. Secondly, a cross-sectional approach was adopted, which is still limited in illuminating the dynamics of the developmental trajectories of preservice EFL teachers’ professional knowledge. Thirdly, this study used primarily tests to assess preservice EFL teachers’ professional knowledge. While the test is designed to reveal preservice teachers’ declarative knowledge of CK, PK, and PCK, it cannot capture or assess their real time teaching practice, which contains a wealth of indicators of their professional knowledge. Therefore, it is highly recommended that classroom observation-based assessment be used together with tests in future research to depict a more comprehensive picture of preservice teachers’ professional knowledge.

Despite these limitations, several valuable implications concerning the sustainable development of preservice EFL teachers’ professional knowledge can be drawn. First, the finding of significant impact of residency practicum under the collaborative supervision of university teachers and school mentors may offer important references to educational policymakers to design high-qualified practicum plans. Second, since our study has shown the close relationship among PK, CK, and PCK, university course designers are expected to recognize the importance of facilitating preservice teachers to integrate these knowledge domains through well-designed courses. Finally, further effort is needed to warrant the quality of classroom observation as a learning opportunity. In particular, teacher educators need to seek effective measures to strengthen the role of classroom observation in promoting the development of preservice teachers’ professional knowledge.

## Data Availability Statement

The raw data supporting the conclusions of this article will be made available by the authors, without undue reservation.

## Ethics Statement

The studies involving human participants were reviewed and approved by Academic Affairs Division of Northeast Normal University. The patients/participants provided their written informed consent to participate in this study.

## Author Contributions

All authors listed have made a substantial, direct, and intellectual contribution to the work and approved it for publication.

## Funding

This work was supported by National Social Science Foundation of China: Tracing the PCK developmental trajectories of full-time M.Ed (Masters of Education). Grant number: BIA170165.

## Conflict of Interest

The authors declare that the research was conducted in the absence of any commercial or financial relationships that could be construed as a potential conflict of interest.

## Publisher’s Note

All claims expressed in this article are solely those of the authors and do not necessarily represent those of their affiliated organizations, or those of the publisher, the editors and the reviewers. Any product that may be evaluated in this article, or claim that may be made by its manufacturer, is not guaranteed or endorsed by the publisher.

## Appendix

Item examples from the test of PK, CK and PCK.

Sample PK Items (20 items).

**Table:**

Sub-dimensions	Item example
Knowledge of teaching structure	The key point of quality education is_________? A. Transmission of knowledge and skills B. Cultivation of innovative spirit and practical ability. C. Cultivation of personality. D. Education of emotion, attitude and values.
Knowledge of general assessment	Which of the following evaluation method is more suitable for examining the knowledge of students’ learning process______? A. Paper-and-pencil test B. Practical operation C. Portfolio assessment D. Questionnaire survey
Knowledge of classroom management	What would you do if the following events occurred in your class room? Please choose one and write your response to the right of the event A. Peter takes Lucy’s paper away and tears it up. B. Jim is repeatedly late for class. C. For the first time, Marry forgets to turn in her homework. D. Steven and Bob get into a fight over whose turn it is as the group leader.
Knowledge of adaptivity	“Let every wall of the school speak.” What is the moral education method used? A. Conversation method B. Method of example demonstration C. Edifying method D. Practice method

Sample CK Items (40 items).

**Table:**

Sub-dimensions	Item example
Knowledge of linguistics	Read the conversation between two people. Answer the questions about their use of language. …. Why does Raquel use “In fact”? A.She is introducing a contrast with what she said earlier. B.She is correcting what Christina said. C.She’s giving herself some time to think. D.She’s marking new point in the story
Knowledge of culture	Both the new and the old editions of the textbook include the text of “Earthquake,” but the new textbook adds a short paragraph at the end of the text. Why is such a paragraph added to this text in the new textbook from the perspective of culture?
Knowledge of literature	What’s the distinctive difference between a play and a short story?

Sample PCK Items (four items).

**Table:**

Sub-dimensions	Item example
Knowledge of students understanding	What difficulties will students encounter when they are learning the past perfect tense and how do you help them solve them?
Knowledge of instructional strategies	If a student says, “The film is interested.” How can you help the student know his mistake and self-correct it?
